# Lightweight strip steel defect detection algorithm based on improved YOLOv7

**DOI:** 10.1038/s41598-024-64080-x

**Published:** 2024-06-10

**Authors:** Jianbo Lu, MiaoMiao Yu, Junyu Liu

**Affiliations:** 1 Guangxi Key Lab of Human-Machine Interaction and Intelligent Decision, Nanning Normal University, Nanning, 530001 China; 2 School of Computer and Information Engineering, Nanning Normal University, Nanning, 530100 China

**Keywords:** Deep learning, YOLOv7, Lightweight network, Strip surface defect detection, D-SimSPPF, Mechanical engineering, Information technology

## Abstract

The precise identification of surface imperfections in steel strips is crucial for ensuring steel product quality. To address the challenges posed by the substantial model size and computational complexity in current algorithms for detecting surface defects in steel strips, this paper introduces SS-YOLO (YOLOv7 for Steel Strip), an enhanced lightweight YOLOv7 model. This method replaces the CBS module in the backbone network with a lightweight MobileNetv3 network, reducing the model size and accelerating the inference time. The D-SimSPPF module, which integrates depth separable convolution and a parameter-free attention mechanism, was specifically designed to replace the original SPPCSPC module within the YOLOv7 network, expanding the receptive field and reducing the number of network parameters. The parameter-free attention mechanism SimAM is incorporated into both the neck network and the prediction output section, enhancing the ability of the model to extract essential features of strip surface defects and improving detection accuracy. The experimental results on the NEU-DET dataset show that SS-YOLO achieves a 97% mAP50 accuracy, which is a 4.5% improvement over that of YOLOv7. Additionally, there was a 79.3% reduction in FLOPs(G) and a 20.7% decrease in params. Thus, SS-YOLO demonstrates an effective balance between detection accuracy and speed while maintaining a lightweight profile.

## Introduction

Strip steel is a significant steel product, that is widely used as a raw material in construction, automobile manufacturing, electric power equipment, shipbuilding and other fields^1^. During the production process, strip steel is susceptible to the influence of various elements, such as the production environment and equipment, resulting in surface defects on the strip steel, including cracks, scratches, pitting, patches, scale, and inclusions. These defects affect the appearance of steel strips and impact the compressive strength, corrosion resistance, and oxidation resistance of the final products^2^. Therefore, detecting surface defects on strip steel is important for improving steel product quality.

With the rapid development of machine vision and deep learning, object detection models based on convolutional neural networks play are vital in strip steel surface defect detection. Defect detection models based on deep learning are primarily classified into two categories: single-stage detection models, represented by SSD (Single Shot Neural Networks)^3^ and YOLO (You Only Look Once)^4–9^, the second category includes R-CNN (Region-Based Convolutional Neural Network)^10^ and Faster R-CNN (Faster Region Convolutional Neural Networks)^11^ algorithms, which are representative of two-stage detection models. Two-stage object detection algorithms perform well in defect detection tasks because of their high detection rate and efficiency. Nevertheless, they often require more computational resources and time, making deployment in practical industrial scenarios relatively difficult. In contrast, single-stage methods do not need to generate candidate regions and can directly predict the object category and position only through the candidate boxes, greatly improving the detection speed and making them highly suitable for real-time strip steel surface defect detection tasks. Yan et al.^12^ improved YOLOv7 with a PConv-based FasterNet module, integrating it into the backbone for enhanced feature extraction and achieving high detection accuracy and speed. Zhou et al.^13^ introduced a precise and efficient detection model, named CABF-YOLO, based on YOLOX for detecting surface defects in strip steel. First, they introduced the TCCA attention module into the backbone of YOLOX to capture the defect positions accurately. Second, a novel Bidirectional Fusion (BF) strategy was used for the neck of YOLOX. The BF strategy enhances the fusion of low-level and high-level semantic information to obtain fine-grained information. Finally, they used the EIoU loss function to accelerate the convergence speed and achieve precise localization. Compared to the original model, CABF-YOLO achieved superior performance. Shen et al.^14^ proposed an algorithm based on YOLOv7 to improve defect detection and enhance the capability of classifying and localizing defects on the surface of hot-rolled strips. The BI-SPPFCSPC structure was incorporated into the feature pyramid in this algorithm, enabling enhanced extraction of features from small objects and improved accuracy in network model positioning. Additionally, a small object detection layer was introduced to enhance shallow feature capture. The mAP of the enhanced YOLOv7 model increased to 80.7%. However, although these developments have improved model accuracy, large model parameters and slow computational speeds remain, which make deploying the models on resource-constrained hardware devices difficult. Therefore, academics have shifted their attention towards methods for achieving model lightweighting. For example, Lang et al.^15^ reduced YOLOv5 parameters by using MobileNetv3 in the neck, replacing SPPF with SE attention in the backbone. The improved model reduced FLOPs by 59.4% and params by 47.9%, with a slight 0.3% decrease in the mAP. Wang et al.^16^ employed GhostNet and achieved both a lightweight model design and guaranteed detection accuracy. After lightweight optimization, the model's parameter count, GFLOPs, and weight distribution decreased by 36.6%, 40%, and 34.7%, respectively. Zhang et al.^17^ proposed a lightweight YOLOv5 strip steel surface defect detection algorithm based on YOLOv5s to balance between detection accuracy and speed. They introduced an efficient, lightweight convolutional layer called GSConv. The Slim Neck, which was designed based on GSConv, replaces the neck in the original algorithm, reducing the number of network parameters and improving the detection speed. While these algorithms have achieved some progress in model lightweighting, a noticeable decrease in accuracy compared to the original model or other lightweight models persist.

To further decrease the model parameters and computational complexity while improving model accuracy and achieving model lightweighting, this paper utilizes the more advanced YOLOv7 model from the YOLO series for lightweight improvement. The proposed SS-YOLO network model aims to balance detection speed and accuracy.

The main contributions of this paper are as follows:

- In this paper, the CBS module in the YOLOv7 backbone network is replaced with the lightweight MobileNetv3 network to reduce model computational complexity while further improving defect detection accuracy. The replacement results in a significant reduction in the computational complexity of only 79.3% of the original model. Additionally, the detection accuracy improves to 96.9%, effectively balancing decreasing model computational complexity and enhancing detection accuracy.
- To expand the target receptive field, enhance the fusion of multi-scale feature information, and significantly decrease the number of network layers and model parameters, this study integrates depth-separable convolution and the SimAM attention mechanism to construct the D-SimSPPF module and replaces the original YOLOv7 module with this module, which has a smaller parameter size than the SPPCSPC module. The number of module parameters decreased by 99.9% after the improvement, essentially realizing the network lightweight.
- The parameter-free attention mechanism SimAM is embedded into the feature fusion module and prediction output module to address the interference caused by noise and other elements in the complicated backdrop of the strip steel production environment. Without increasing the network parameters, a better integration of features extracted from the feature extraction network is achieved by adaptively learning weight parameters and capturing more detailed information. This effectively enhances the detection accuracy for surface defects on strip steel targets.

The subsequent sections of this paper are structured as follows. Section 2 presents a literature review. Section 3 provides a comprehensive explanation of the proposed SS-YOLO model for steel strip surface defect detection. Section 4 evaluates the experimental results and compares our proposed model with cutting-edge techniques. Section 5 concludes this paper.

## Related works

The traditional visual detection process is usually divided into three stages: region proposal, feature extraction, and feature classification. The purpose of region selection is to identify potential targets in certain regions of the input image. Conventional approaches frequently employ numerous cycles of scanning the input image using sliding windows of various sizes to acquire potential regions^18^. Representative features are retrieved from the candidate region images via feature extraction methods such as Local Binary Pattern (LBP), Histogram of Oriented Gradients (HOG), and Gray-Level Co-occurrence Matrix (GLCM). These features are represented as feature vectors. These feature vectors serve as input for feature classifiers, such as Support Vector Machines, decision trees, and multi-layer perceptrons, in the third stage, which are used for defect classification. Lou et al.^19^ suggested that defect classification is indispensable to defect detection, serving as a crucial prerequisite for achieving online quality inspection of final products. They conducted a focused and systematic review of both traditional and emerging computer vision-based automated defect classification methods. Zhao et al.^20^ used IPCA to compress each frame image and extract effective features. The projection coefficients of each frame image on the principal components were input into an SVM model for classification, facilitating surface defect detection in steel-strip products. Duan et al.^21^ utilized a novel two-stream convolutional neural network to extract features from original RGB images and gradient images for the first time. Afterwards, the wavelet transform was used for feature fusion, which was then fed into the SVM classifier to classify and identify the surface defects of the aluminum profiles. Chu et al.^22^ proposed a surface defect detection algorithm that utilizes enhanced SVM and multi-type statistical methods and achieved a recognition rate of 94% for five types of steel surface defects. Zhang et al.^23^ proposed a method that combines fitting the histogram and membership matrix of test images with Gaussian functions to locate and detect defects. Hu et al.^24^ employed a genetic algorithm to optimize a surface defect classification model for strip steel by constructing an SVM classifier, enhancing defect classification accuracy. However, these traditional defect detection methods exhibit limited robustness when handling defects of different types and sizes. With to the advancement of deep learning technology, an increasing number of defect detection tasks have shifted towards the use of deep learning methods such as Convolutional Neural Networks (CNN). These methods can automatically learn higher-level feature representations, reducing reliance on manual feature design and providing powerful means for achieving more refined quality control in manufacturing.

In recent years, many researchers have combined deep learning frameworks with defect detection methods, developing end-to-end defect detection network models and achieving significant results. Bai et al.^25^ proposed an improved defect segmentation network with a coder-decoder structure to realize muti-scale interaction of features. The experimental results confirm that an IoU of 89.11% and a Dice coefficient of 94.24% can be achieved for the Severstal dataset using this method. Jens et al.^26^ proposed a new big-data handling paradigm for quality monitoring and improvement in flat steel production based on the efficient exploitation of high resolution (HR) measuring data. Luo et al.^27^ proposed a decoupled two-stage object detection framework based on CNNs to address the challenges in detecting surface defects on flexible printed circuit boards (FPCB). To effectively locate non-salient defects, a multi-hierarchical aggregation (MHA) block is proposed as a location feature (IF) enhancement module in the defect localization task. Moreover, to accurately classify similar defects, a locally non-local (LNL) block is presented as an SEF enhancement module in the defect classification task. Chen et al.^28^ proposed a genetic algorithm-based Gabor faster region-based CNN (Faster GG R-CNN), which incorporates Gabor kernels into Faster R-CNN. They devised a two-stage training methodology that combined a genetic algorithm (GA) and backpropagation to train the Faster GG R-CNN model, enabling effective textile defect detection under varying backgrounds, positions, and sizes. Shi et al.^29^ proposed an improved Faster R-CNN network for steel surface defect detection, in which the ConvNeXt structure replaced the original backbone network, and the k-means clustering algorithm was utilized to generate anchors more suitable for target defects. The proposed method achieved an improved 8.4% over the original network on the NEU-DET dataset.

YOLO is a leading single-stage algorithm in deep learning object detection methods. The fundamental revolves around partitioning the input image into numerous grids and forecasting the category and position of the object for each grid. Starting from YOLOv1 and progressing to YOLOv8, its performance has continuously improved, gradually reaching an equilibrium between detection speed and accuracy^4–9^. This enables YOLO to identify minor imperfections on the surface of steel strips more accurately. Consequently, a multitude of superior algorithms for detecting surface defects on steel strips have been suggested, utilizing the YOLO technique as a foundation. Wang et al.^30^ enhanced strip steel defect detection using YOLOv7. They integrated ConvNetXT into the backbone for faster inference and replaced YOLOv7's original ELAN module with the improved C3 module in the head, streamlining the network. The improved model achieved 82.9% accuracy. Gao et al.^31^ proposed SRN-YOLO for steel surface detection based on YOLOv7 to accurately detect small and blurry target defects in steel defect detection. First, they designed a split residual convolution network (SResNet) to capture gradient feature information. Then, a refusion feature pyramid network (RFPN) is built to minimize the loss of features. Furthermore, to increase the number of positive samples of small targets, they combined the Normalized Wasserstein Distance (NWD) and Complete Intersection Over Union (CIOU). The experimental results show that SRN-YOLO reaches an mAP of 81.2% on the NEU-DET dataset. Xie et al.^32^ proposed a detection algorithm based on the improved YOLOv7 algorithm for steel surface defect detection. First, they combined a transformer module and InceptionDWConvolution to increase the ability of the network to detect small objects. Second, the SPPFCSPC structure is introduced to enhance the network training performance. To optimize the network structure and improve model performance, the GAM attention mechanism and Mish function were integrated. The experimental results show that on the Northeastern University Defect Detection (NEU-DET) dataset, the improved YOLOv7 network model improves the mean average precision (mAP) by 6% compared to that of the original algorithm. Zhao et al.^33^ developed the stem module and combined it with the MobileNetv2 module with a CA attention mechanism and integrated it into the backbone network of YOLOv5 to decrease the number of model parameters and increase the detection speed. The speed of detecting objects with the LSD-YOLOv5 model increased by 28.7%. Wang et al.^34^ replaced the ELAN module in the YOLOv7 model with bottleneck transformer 3, improving model accuracy while making the network model more lightweight. Li et al.^35^ first developed ResNet-Mini to pre-classify the original dataset, reducing the computational burden. Then, they conducted image preprocessing to enhance defect features. Subsequently, they applied OTSU and normal distribution enhancement algorithms to extract feature grayscale. Finally, they used the improved YOLOv5s model to detect surface defects on strip steel. While the aforementioned algorithms have made notable contributions to improving detection accuracy, minimizing model parameters, and accelerating detection speed, a satisfactory balance has not yet been achieved among detection accuracy, speed, and model parameter count. Therefore, to further improve detection precision while maintaining high-speed operation, this paper proposes the augmented SS-YOLO network model based on YOLOv7.

In 2022, Wang et al.^9^ presented the YOLOv7 model, which integrated strategies such as structural reparameterization, positive and negative sample allocation, and a training approach with an auxiliary head, achieving a favourable trade-off between detection efficiency and accuracy. YOLOv7 exhibits substantial enhancements in performance compared to previous iterations of YOLO models, outperforming alternative object detection algorithms in terms of both detection speed and accuracy. Nevertheless, to adjust to identifying surface flaws on steel strips in production workshops, improving the YOLOv7 model is crucial. High-speed operation should be ensured while further improving strip surface defect detection accuracy.

## Methods

### Network framework

**Figure 1:**
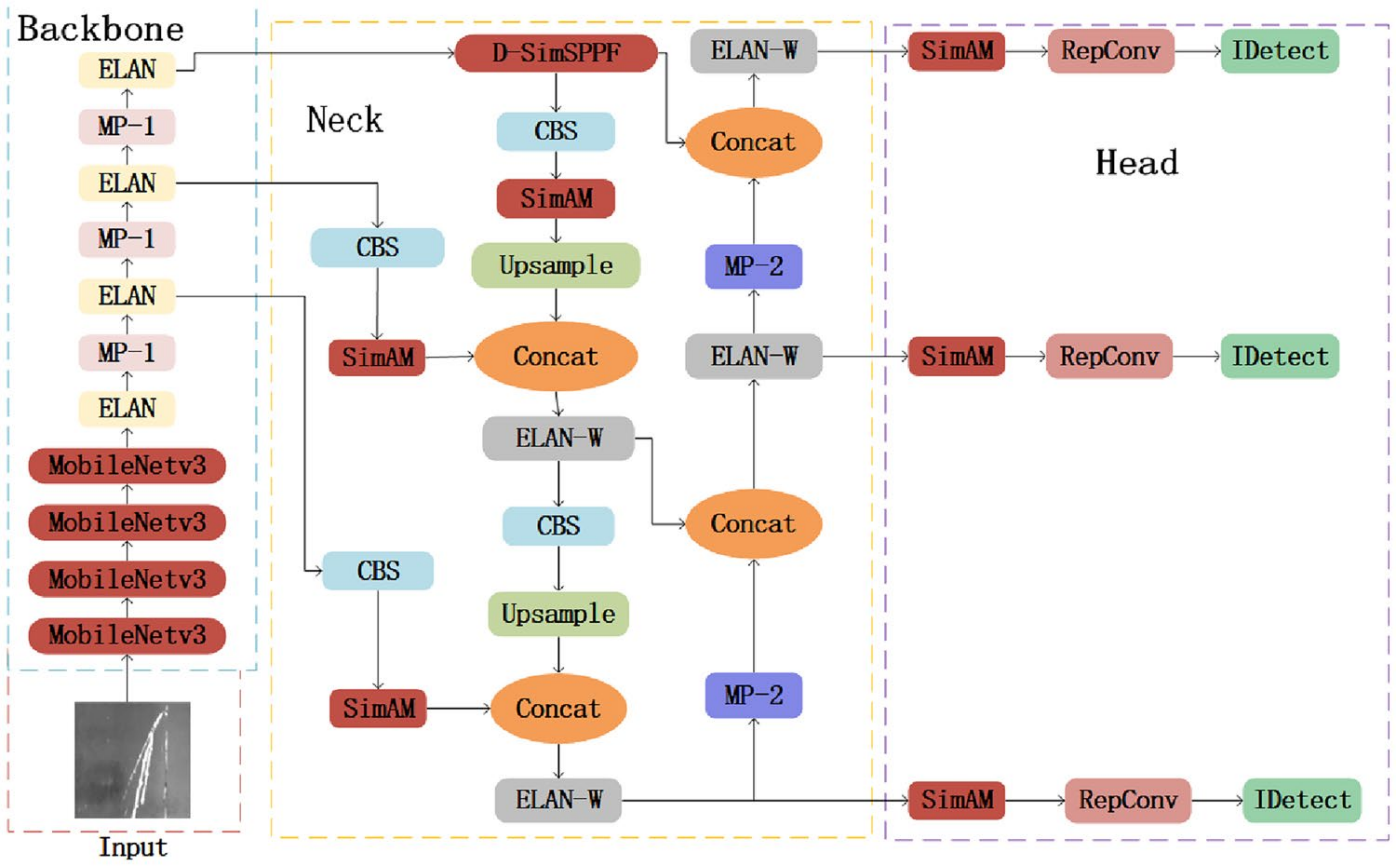
SS-YOLO network structure.

Despite YOLOv7’s excellent capabilities in object detection and its proven effectiveness in several applications, its deployment for real-time detection of surface defects on steel strips is limited by its numerous model parameters, high computational complexity, and slow detection speed. This is challenging when deploying on hardware devices with limited resources. In response to these issues, the SS-YOLO model is built upon the YOLOv7 model for lightweight improvement. The SS-YOLO network structure is shown in Fig. 1, with specific enhancements in the YOLOv7 structure emphasized in red boxes in this paper.

The SS-YOLO network comprises the input, backbone, neck, and head layers. The input adopts adaptive image scaling and mixed data augmentation techniques to ensure that various input images are scaled to a uniform size, thereby meeting the requirements of the backbone network for input sizes. The backbone network comprises three parts, MobileNetv3, E-ELAN, and MP-1, which are used for feature extraction to ensure the completeness of feature extraction. The SS-YOLO network replaces the CBS module in the original YOLOv7 network with the lightweight network MobileNetv3 to compress model parameters and improve detection speed. In the SS-YOLO neck, the D-SimSPPF module is used to replace the SPPCSPC module in the original YOLOv7, further expanding the receptive field of the backbone network, enhancing feature information fusion, further reducing network layers, minimizing model parameters, and reducing inference time. The neck uses the PAFPN (Path Aggregation Feature Pyramid Network) structure to integrate low-level spatial information and high-level semantic features. By combining bottom-up feature extraction with top-down feature fusion, features from different scales are fused. Following this, features corresponding to objects of three distinct scales—large, medium and small—are directed into the head. After passing through the RepConv module, bounding boxes for the targets are generated, and their categories are predicted, resulting in the final prediction outcomes. Furthermore, the parameter-free SimAM attention mechanism is integrated into both the feature fusion and the prediction output. SimAM can fulfil the criteria for designing lightweight models while accounting for spatial and channel dimensions. This strengthens the fusion capability of target features across various dimensions, resulting in increased accuracy in detecting surface flaws on steel strips. The improved SS-YOLO model balances between detection speed and accuracy.

### MobileNetv3-based backbone network improvements

CNN-based deep learning networks typically employ many convolutional operations to extract feature information from images and are extensively utilized in target detection tasks. However, deploying neural network models on mobile and embedded devices remains a significant challenge due to limited storage space and computational capability. Hence, decreasing the computational complexity and parameter count of the model while lightweighting the model is imperative to meet the demands of real industrial scenarios.

The backbone feature extraction network of the YOLOv7 model is built upon CSPDarknet53^9^. Although the model has strong feature extraction capabilities, it requires many parameters, which leads to a significant increase in computational workload. To increase the computational speed of the model in real detection applications, the lightweight MobileNetv3 network is used as the backbone feature extraction network for YOLOv7. This decision aims to decrease the number of parameters and computational burden, resulting in a higher detection speed. More precisely, the CBS module of the YOLOv7 backbone network is replaced with MobileNetv3^36^. The MobileNetv3 network combines depth-separable convolutional operations, which effectively decreases the computational burden and makes it more compatible with resource-limited mobile devices, in contrast to the conventional CBS.

**Figure 2:**
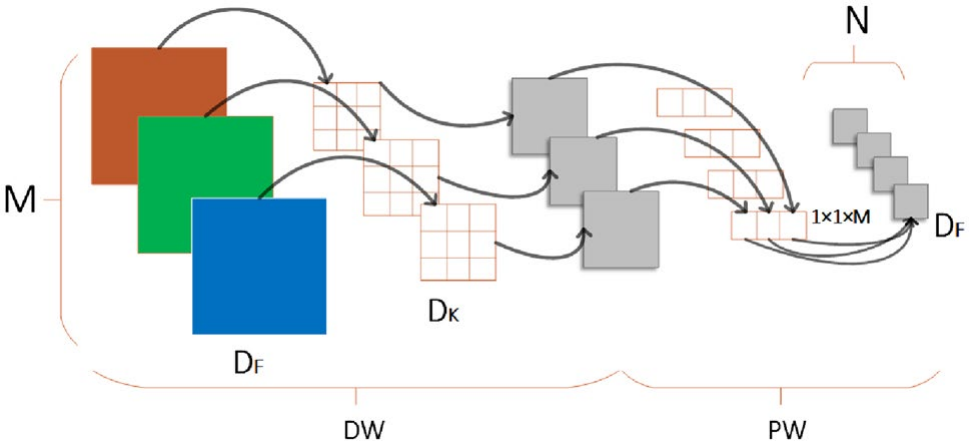
The realization of depthwise separable convolution.

DepthWise Convolution (DWC) is the main feature of the MobileNet series.In the Depthwise Separable Convolution operation, the Depthwise Separable Convolution operation utilizes Depthwise Convolution (DW) and Pointwise Convolution (PW) to extract features from images. Figure 2 depicts the network architecture.

The depthwise convolution applies M convolution kernels of size D_K_ × D_K_ × 1 to the feature map of size D_F_ × D_F_ × M to generate M feature maps. Subsequently, the feature maps are fed into the pointwise convolution, where convolution is performed using N kernels of size 1 × 1 × M. Ultimately, obtain the output features of size D_F_ × D_F_ × N are obtained. The computational quantity Q_D_ and parametric quantity P_D_ are represented by Eqs. (1) and (2) respectively.

1 $${\text{Q}}_{{\text{D}}} = {\text{D}}_{{\text{F}}} \times {\text{D}}_{{\text{F}}} \times {\text{M}} \times {\text{D}}_{{\text{K}}} \times {\text{D}}_{{\text{K}}} + {\text{M}} \times {\text{N}} \times {\text{D}}_{{\text{F}}} \times {\text{D}}_{{\text{F}}}$$

2 $${\text{P}}_{{\text{D}}} = {\text{D}}_{{\text{K}}} \times {\text{D}}_{{\text{K}}}\times{\text{M}} + {\text{M}} \times {\text{N}}$$

Equations (3) and (4) display the formulas for the conventional convolutional computational quantity Q_R_ and the parametric quantity P_R_.

3 $$Q_{R} = D_{K} \times D_{K} \times D_{F} \times D_{F} \times M \times N$$

4 $$P_{R} = D_{K} \times D_{K} \times M \times N$$

The equations representing the ratio between the computational volume of the depth separable convolution and the ordinary convolution, denoted as R_Q_, and the ratio of the parametric volume, denoted as R_P_, are presented in equations Eqs. (5) and (6).

5 $$R_{Q} = \frac{{Q_{D} }}{{Q_{R} }}=\frac{1}{{D_{K} \times D_{K} }} + \frac{1}{N}$$

6 $$R_{P} = \frac{{P_{D} }}{{P_{R} }} = \frac{1}{{D_{K} \times D_{K} }} + \frac{1}{N}$$

Compared to standard convolution, depthwise separable convolution significantly reduces the number of parameters and computations, greatly increasing the model speed and reducing the computational cost. Substituting MobileNetv3 for standard convolution in the backbone network not only saves computational expenses for strip surface defect detection but also decreases the number of model parameters and improves the detection speed.

### D-SimSPPF module

The SPPCSPC module in the YOLOv7 feature fusion network is constructed using the SPP module as its foundation^9^. The suggested strategy incorporates the features of the SPP module for multi-scale spatial pyramid pooling of input features. Additionally, it improves the representational capacity by extracting feature information at multiple scales using parallel convolution and maximum parallel pooling kernels. Although the SPPCSPC module performs well, its large number of parameters and higher computational cost, in turn, affect the network inference speed. Thus, we propose D-SimSPPF, which utilizes the design concept of SimSPPF (Spatial Pyramid Pooling Fusion) module to achieve fewer parameters and faster speed.

The SimSPPF module optimizes the parallel pooling operation in the SPP module. The input is partitioned into two parts, with one part being linked in a sequential manner to three maximum pooling layers (Max Pooling) of size 5 × 5. The remaining portion is directly concatenated with the output of the three pooling operations, resulting in features of a predetermined size, which greatly expands the receptive field and enhances the features. Moreover, the SimSPPF module substitutes SiLU with ReLU as the activation function, optimizing the processing speed^8^.

**Figure 3:**
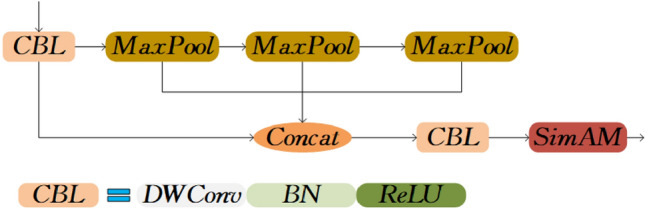
D-SimSPPF module structure.

However, despite utilizing the SimSPPF module to enhance the feature fusion network, the model retains an elevated parameter count, with a predominant concentration on the 1 × 1 standard convolution. To further minimize the number of model parameters and increase the detection efficiency, we substitute the ordinary convolution in the CBL module with depthwise separable convolutions. This modification enables the network to more effectively integrate multi-scale feature information, lower floating-point operations, decrease model parameters, and increase inference speed. However, the lightweight design has a certain adverse impact on the detection accuracy. Consequently, we introduce the parameter-free SimAM attention mechanism in the SimSPPF module, which enables the model to focus more on relevant feature information without introducing extra parameters, thus minimizing attention to other unimportant information. The structure of the D-SimSPPF module is depicted in Fig. 3.

### Integration of SimAM attention mechanisms

To address the interference caused by factors such as noise in the complex background of strip steel production environments, and to increase model detection accuracy while guaranteeing model lightweighting, the SimAM(Simple Parameter-free Attention Moudle) was introduced^37^. In contrast to current channel attention and spatial attention mechanisms, the SimAM attention mechanism can simultaneously focus on the importance of both channel and spatial features. By maintaining the same network parameters, the three-dimensional weights of feature mapping can be deduced. This raises the focus of the network on surface defects in strip steel, boosts the detection capability of significant objects, and inhibits the interference caused by complex backgrounds. Consequently, surface defect detection accuracy on the strip is increased. The parameter-free attention mechanism is based on neural science theory and assigns a unique weight to each neuron. The expression of its principle is shown in Eq. (7).

7 $$\tilde{X} = {\text{sigmoid}}\left( \frac{1}{E} \right) \otimes X$$

In the Eq. (7), E represents grouping across channel and spatial dimensions. The sigmoid activation function limits excessively large values of E, ensuring that they do not impact the relative importance of each neuron. The input features X belong to the space C × H × W.

**Figure 4:**
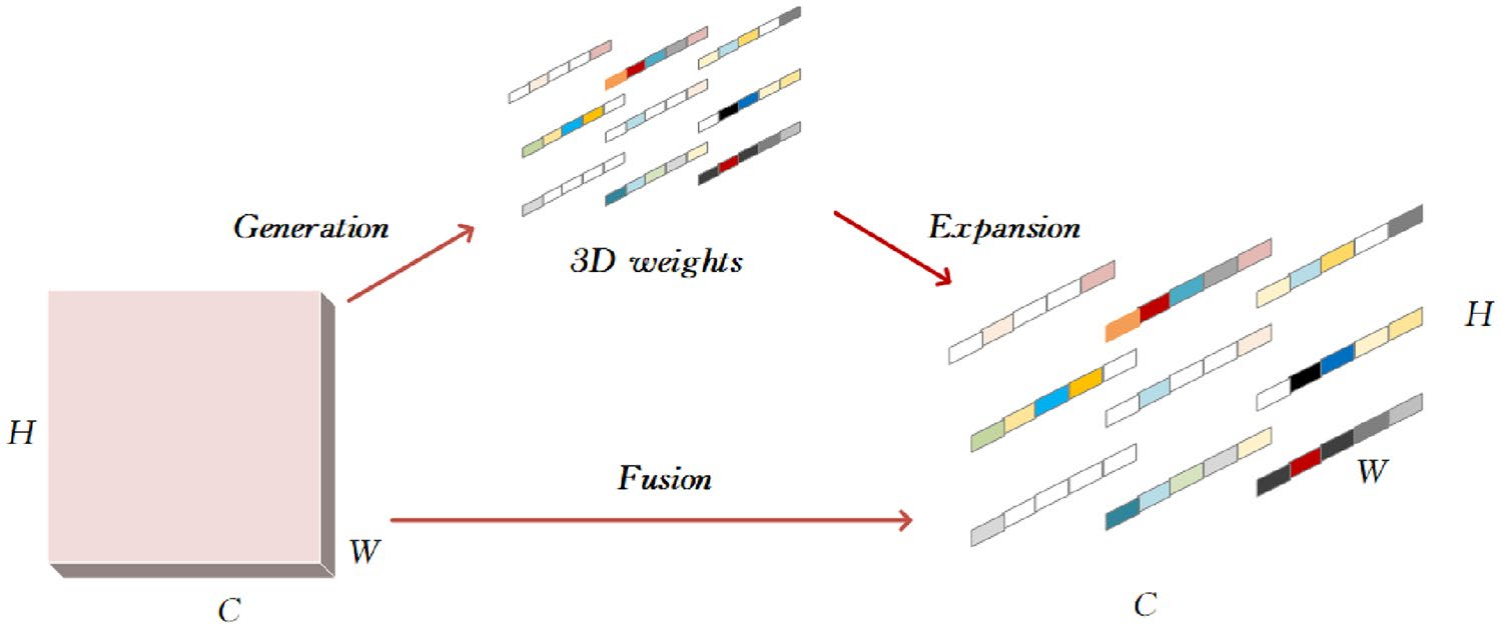
SimAM network structure. SimAM can derive 3D attention weights for feature maps without introducing additional parameters. After obtaining the weights for each neuron, it directly evaluates the importance of independent neurons and assigns higher weights to important neurons, enabling the model to focus on surface defect features of steel strips and enhance detection performance.

This paper applies the SimAM attention mechanism to a feature fusion network. It optimizes two effective feature layers extracted from the backbone network, enhances the neuron output of multi-scale information, suppresses the interference of irrelevant features from adjacent neurons, and focuses closely on the deep feature information of the strip steel surface defects. In addition, the SimAM attention mechanism is integrated prior to the subsequent feature fusion network executing the up-sampling operation to guarantee the capture of more distinctive feature information during the up-sampling process, improving the ability of the network to detect defective targets. Prior to the outputting detection results for the three scales, the SimAM attention mechanism is combined to improve the impact of the head section on the object detection outcomes. The SimAM attention mechanism is introduced to increase the impact weight of the head section on the object detection results. A schematic diagram of the principle of the SimAM attention mechanism is shown in Fig. 4.

## Experiments

### Experimental environment and setup

The experimental parameters for this experiment are as follows: the image resolution is set to 640 × 640, the batch size is set to 16, and the number of epochs is set to 300. The YOLOv7 model is used as the baseline model and the yolov7.pt weights are utilized for pre-training the model. The experimental environment configuration is shown in Table 1.

**Table 1:** Experimental environment.

Environment	Details
Operating system	Windows 10
CPU	Intel(R)i5-12,400
GPU	NVIDIA RTX3060
Deep learning framework	PyTorch1.6.0

### Datasets and data enhancement

**Figure 5:**
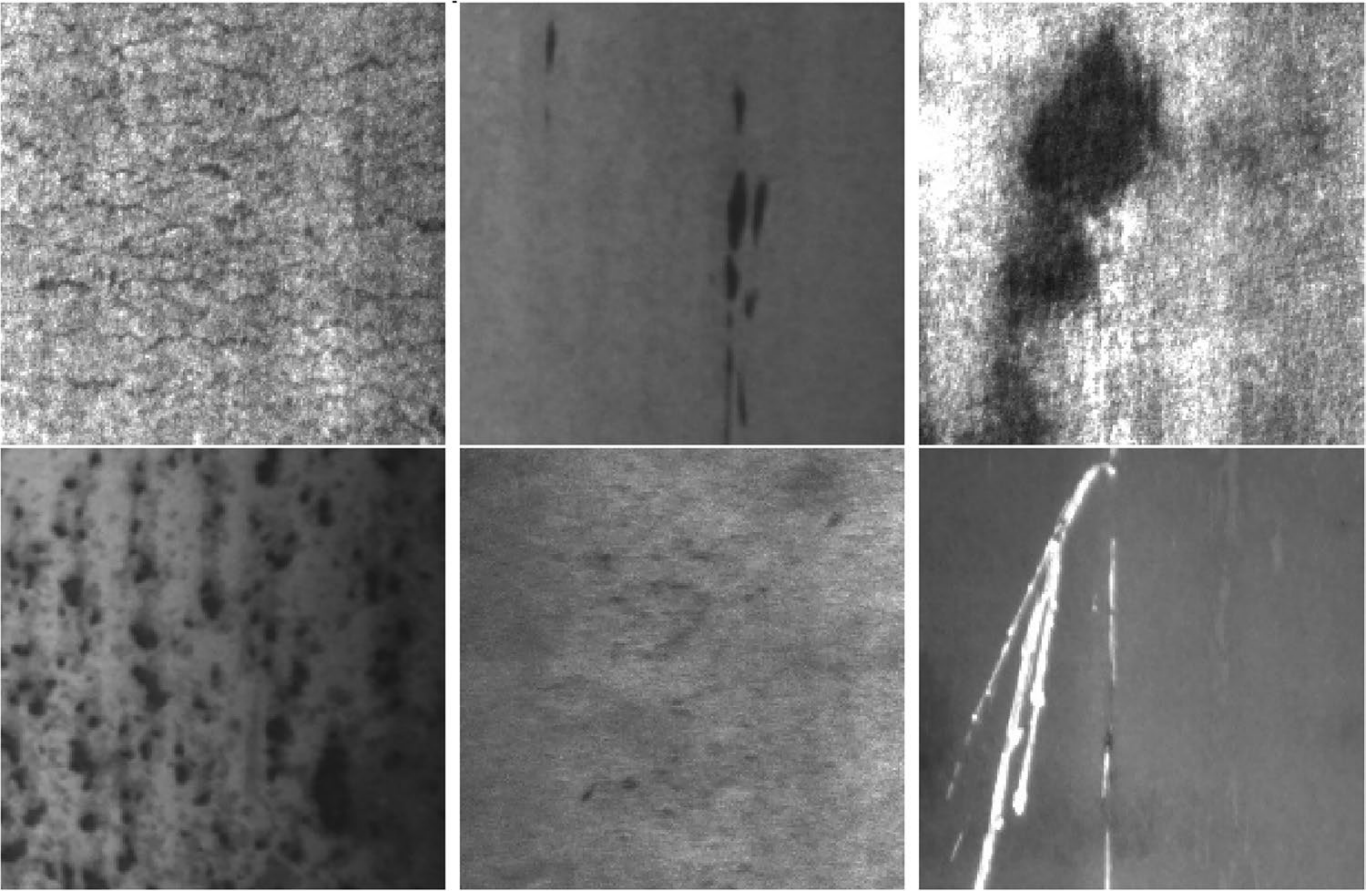
NEU-DET surface defects.

The experiments in this paper utilized the NEU-DET dataset, which consists of surface defects on strip steel and was released by Northeastern University^38^. The dataset comprises six distinct categories of hot-rolled strip surface defects: crazing (Cr), inclusion (In), patches (Pa), pitted_surface (PS), rolled-in scale (RS), and scratches (Sc). The NEU-DET dataset contains 300 images for each type of defect, resulting in a total of 1800 images. Each image has a size of 200 × 200 pixels. The NEU-DET surface defects dataset is displayed in Fig. 5.

**Figure 6:**
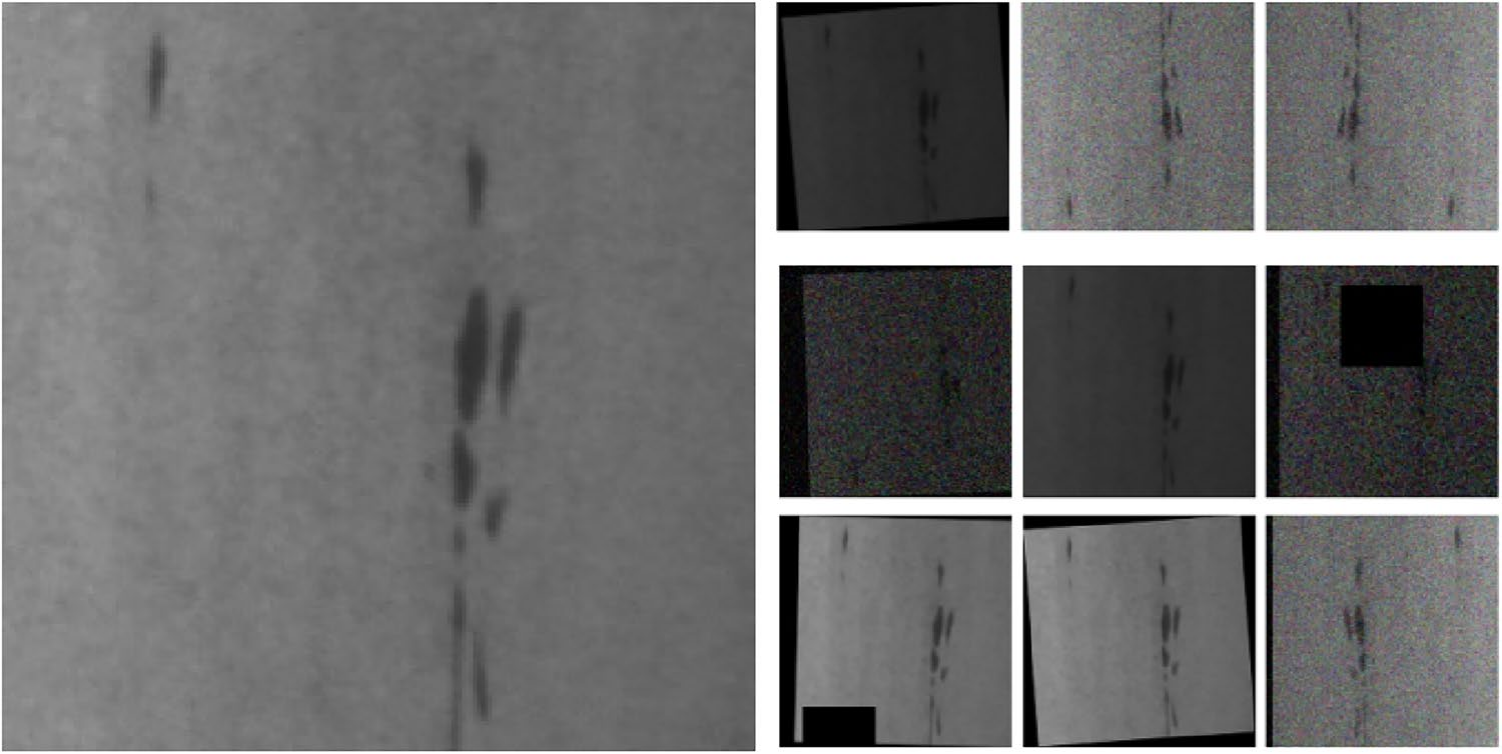
Illustration of the enhancement effect.

By organizing the data, it was noted that each category within the NEU-DET dataset involved relatively few images. Nevertheless, a limited dataset can result in network overfitting. Therefore, this experiment uses Python language augment the size of the dataset, mainly using Crop, Pan, Change Brightness, Add Noise, Rotate Angle, Mirror, and Cutout change operations to pre-augmented the dataset up to 18,000 images. Additionally, the experiment randomly generated the training, test, and validation sets at an 8:1:1 ratio. This aids in preventing network overfitting and strengthens the generalizability and robustness of the network. Figure 6 illustrates several examples of the enhancing effect.

### Evaluation metrics

This experiment focuses on evaluating the strip steel defect detection model by focusing on two key factors: detection accuracy and speed. The performance of the steel-strip defect detection model is evaluated using Average Precision (AP), mean Average Precision (mAP), Floating-Point Operations (FLOPS), and Parameters (Params). The AP metric quantifies the average detection precision for each defect category. It is related to the accuracy P and recall R. mAP represents the average detection precision of all defect categories, which serves as an indicator of the overall accuracy of the model. The number of FLOPs reflects the total number of floating-point operations required for the implementation of the algorithm, which is used as a measure of the computational complexity of the algorithm. Lower FLOPs (G) typically signifiy a lower computational complexity, implying that the algorithm can complete computations in a relatively short time, thus realizing real-time detection. The specific expressions for calculating these metrics are as follows in Eqs. (8)–(11).

8 $$P = \frac{TP}{{TP + FP}}$$

9 $$R = \frac{TP}{{TP + FN}}$$

10 $$AP = \int_{0}^{1} P ({\text{r}})dr$$

11 $$mAP = \frac{{\sum_{i = 1}^{c}AP(i) }}{c}$$

In the evaluation metrics, *P* represents precision; TP denotes the number of accurately identified faulty samples; FP indicates the number of non-defective samples that are erroneously identified as defective samples; *R* represents recall; FN reflects the number of defective samples that were not correctly detected; AP represents the region bounded by the *P*–*R* curve; mAP signifies the mean detection accuracy across all categories, and a higher value indicates superior model detection ability.

### Analysis of the experimental results

**Figure 7:**
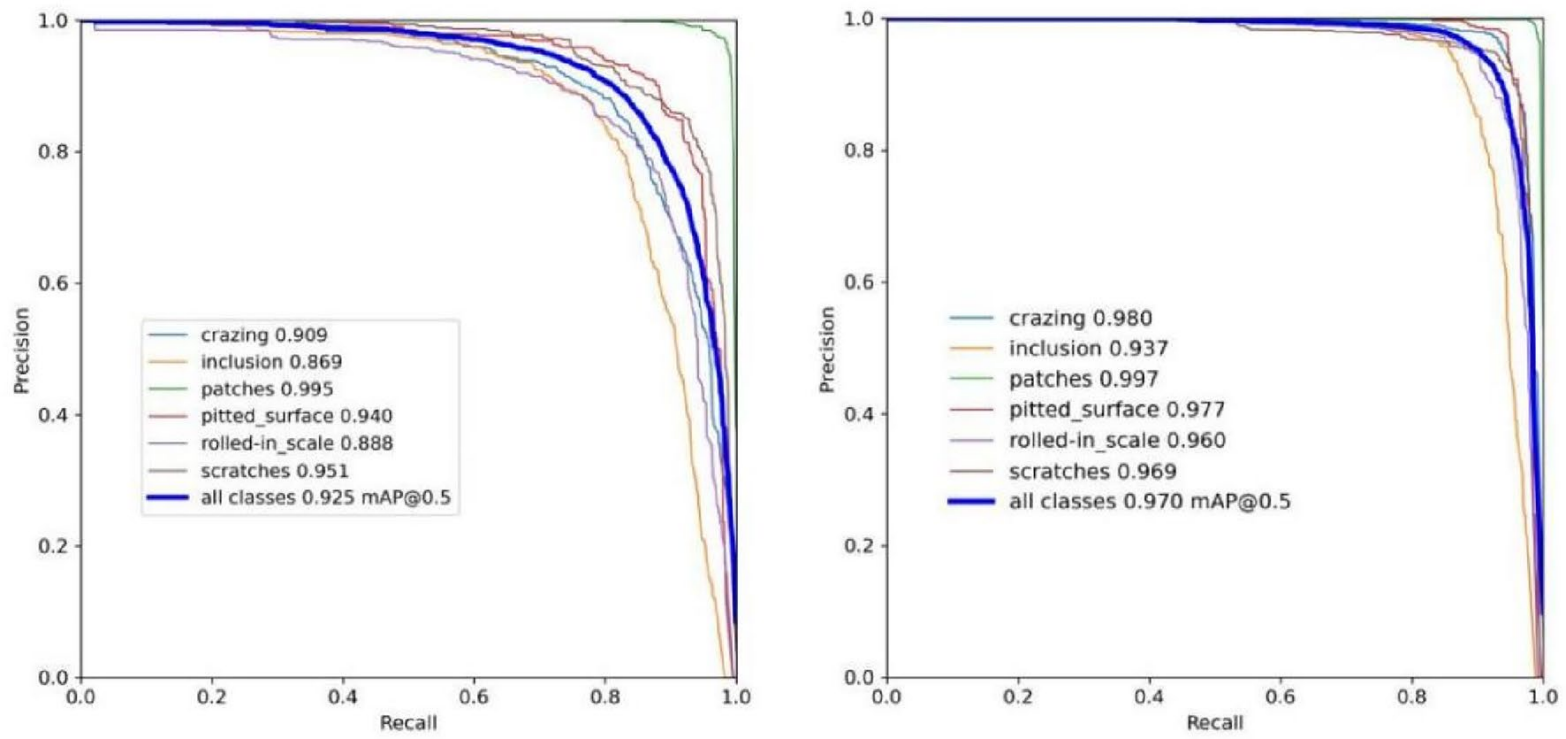
Comparison of the YOLOv7 and SS-YOLO model training results.

To evaluate the effectiveness of our model, we trained both the original YOLOv7 algorithm and the enhanced algorithm (SS-YOLO) on the NEU-DET dataset. This demonstrates the performance of SS-YOLO while keeping the parameter indices unchanged. The enhanced SS-YOLO model achieves an mAP of 97%, representing a notable improvement of 4.5 percentage points compared to YOLOv7. The left image shows the training results of YOLOv7, while the right image displays the training results of SS-YOLO. A comparison of the training results is displayed in Fig. 7.

**Figure 8:**
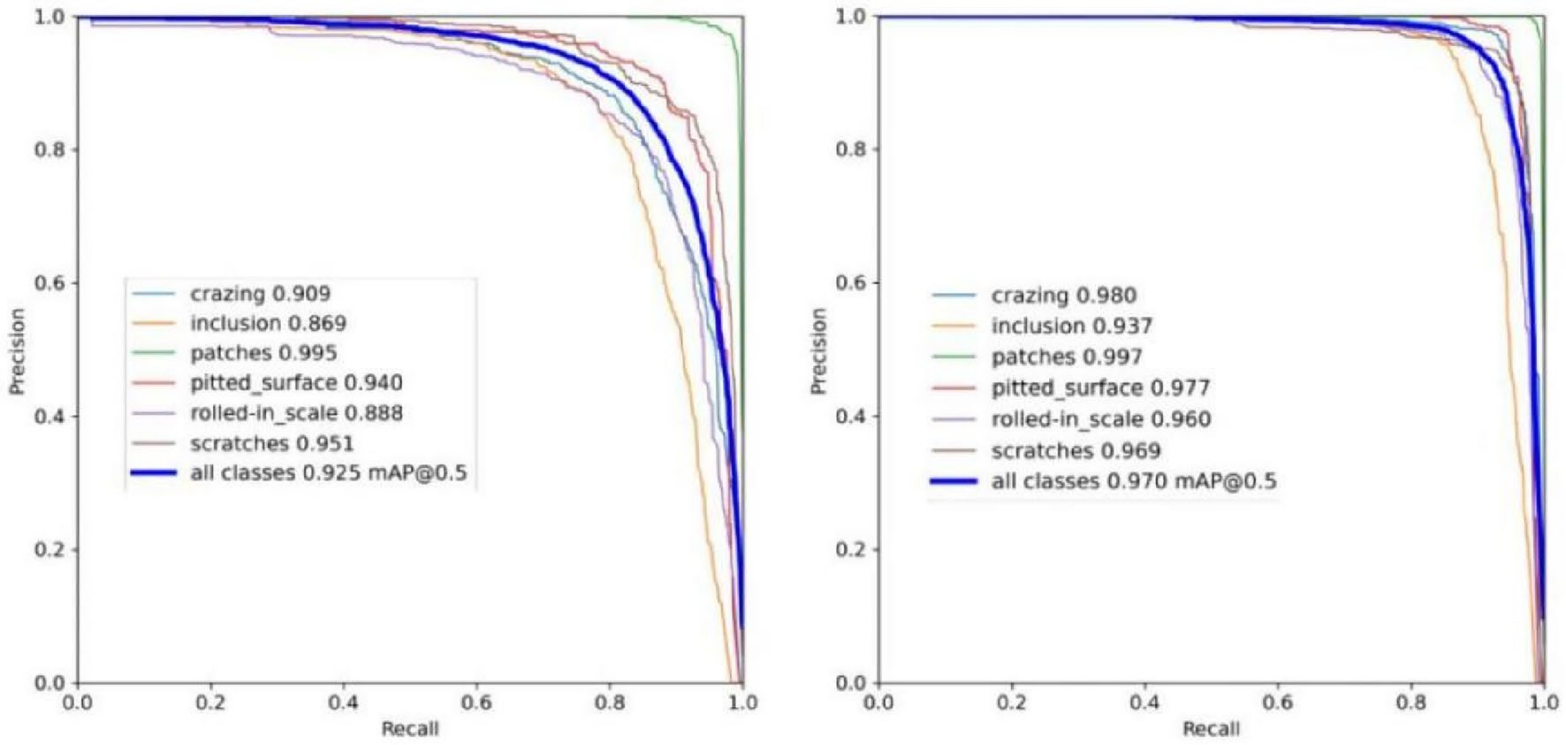
Comparison of YOLOv7 and SS-YOLO Precision-Recall (P-R) curves.

The Precision-Recall (P-R) curve is a crucial metric of model performance. A greater region encompassed by the P-R curve indicates superior performance. Figure 8 displays the Precision-Recall curves of the original YOLOv7 and modified SS-YOLO model for detecting six faults in the NEU-DET dataset. Figure 8 demonstrates that the SS-YOLO algorithm (right), introduced in this paper, outperforms the original YOLOv7 (left) in terms of detection performance.

To further confirm the effectiveness of our proposed SS-YOLO model in detecting surface defects on steel strips, we selected the Params, FLOPs (G) and mAP indicators for comparison it with the YOLOv7 and the SS-YOLO. The results of this comparison can be found in Table 2.

**Table 2:** Comparison of model performance.

Model	Params (M)	FLOPs (G)	mAP (%)
YOLOv7	37.2	105.2	92.5
SS-YOLO	29.5	21.8	97

Table 2 shows that YOLOv7 has 37.2 M parameters, 105.2 FLOPs (G), and a 92.5% mAP. While demonstrating good accuracy, room for improvement in parameters and computational complexity remains. Compared with YOLOv7, the improved SS-YOLO model further optimizes the parameters and complexity, reducing the number of parameters by 7.7 M and the complexity by 83.4 FLOPs (G). It achieves a 97% mAP, significantly improving the detection speed and accuracy. Overall, the algorithm exhibits exceptional precision in identifying surface defects on steel strips, making it an excellent lightweight network design solution with high practical value for deployment on edge devices.

### Comparison of attention mechanisms

In this section, we conducted an attention mechanism comparison experiment on the NEU-DET dataset to validate the effectiveness of the SimAM attention mechanism. The trials were conducted without altering the training parameters. The YOLOv7 algorithm incorporates many attention processes, such as SE^39^, ECA^40^, CA^41^, CBAM^42^, and SimAM^37^. We conducted a comparative analysis of several attention strategies to assess their impact on the model's detection effectiveness. The results of the attention mechanism comparison experiment are presented in Table 3.

**Table 3:** Attention mechanisms comparative experiment.

Model	Params	FLOPs (G)	mAP (%)	AP%					
				Cr	In	Pa	Ps	Rs	Sc
YOLOv7	37,223,526	105.2	92.5	90.9	86.9	99.5	94	88.8	95.1
YOLOv7 + ECA^40^	37,223,526	105.2	92.5	91	87.5	99.5	94.4	88.9	94
YOLOv7 + CBAM^42^	37,320,574	105.2	92.8	91.0	87.5	99.5	94.0	89.5	95.4
YOLOv7 + CA^41^	37,304,918	105.3	93	91.3	88	99.4	94.2	89.3	96
YOLOv7 + SE^39^	37,319,782	105.3	93.2	91.6	88.4	99.4	94.6	90.1	95.3
YOLOv7 + SimAM^37^	37,223,526	105.2	93.1	91.8	87.8	99.6	94.2	90.1	95.6

The experiments indicate that integrating the SE, CA, and CBAM attention mechanisms into YOLOv7 increases the mAP accuracy. However, this improvement comes with increased parameters and computational demand, making these mechanisms unsuitable for optimizing a lightweight strip steel defect detection model. Nevertheless, combining the ECA and SimAM attention mechanisms does not increase the number of model parameters or the computational complexity. This makes them suitable for hardware-constrained devices. Additionally, SimAM significantly improved the mAP by 0.6%, surpassing that of ECA for surface defect detection on steel strips. A comparison of the experimental results shows that employing the SimAM attention mechanism allows for more effective extraction of particular features from the target region. This improvement occurs without adding additional model parameters, enhancing the accuracy of detecting surface defects on steel strips. Ultimately, SimAM effectively strengthens the model.

### D-SimSPPF module performance comparison

To assess the effectiveness of the lightweight module D-SimSPPF, we compared its performance with that of the SPPCSPC module and the SimSPPF module. The comparative experimental results are presented in Table 4. Table 4 demonstrates that the D-SimSPPF module has enhanced performance in terms of Parameters, Inference Times, and FLOPs (G).

**Table 4:** Performance comparison of the D-SimSPPF module.

Models	Params	Inference Times (ms)	FLOPs (G)
SPPCSPC	7,609,344	42	23.3
SimSPPF	1,574,912	11.3	22.4
D-SimSPPF	5120	5.5	21.8

The analysis of the data in Table 4 clearly demonstrates that replacing the SPPCSPC module with the SimSPPF module leads to a significant reduction of 79.3% in module parameters, a decrease of 0.9 M in FLOPs (G), and an increase of 30.7 ms in inference speed.

Due to the substantial number of parameters in the SimSPPF module, deep separable convolution and the SimAM attention mechanism were incorporated on the basis of the SimSPPF module to further enhance module performance, resulting in the proposed D-SimSPPF module. The comparative data show that the D-SimSPPF module parameters decreased to 5120, the inference speed increased to 5.5 ms, and the FLOPs (G) decreased to 21.8. It is evident that the substitution of the SPPCSPC module with the D-SimSPPF module results in a conspicuous reduction in model parameters and an acceleration in detection inference time. This module effectively fulfils the lightweight criteria for detecting surface defects on steel strips, demonstrating the effectiveness of this module improvement.

### Ablation experiment

In this section, ablation experiments are conducted on the NEU-DET dataset using YOLOv7 as the baseline model. Successive experiments were performed by incorporating MobileNetv3, the D-SimSPPF module, and the SimAM attention mechanism into the YOLOv7 network. The symbol “√” indicates method utilization, and the results are presented in Table 5.

**Table 5:** Ablation experiment.

Model	YOLOv7	MobileNetv3	D-SimSPPF	SimAM	Params	FLOPs (G)	mAP (%)
Method 1	√	–	–	–	37,223,526	105.2	92.5
Method 2	√	√	–	–	37,105,506	23.3	96.9
Method 3	√	√	√	–	29,501,282	21.8	96.7
Method 4	√	√	√	√	29,501,282	21.8	97

Table 5 shows, that Method 1 represents the experimental results of the YOLOv7, which serves as the baseline for the following three sets of experiments.

In Method 2, the MobileNetv3 lightweight backbone network is combined with the feature extraction network of YOLOv7. Compared to the data of Method 1, replacing the CBS module of the YOLOv7 backbone network with the MobileNetv3 network leads to a reduction in the number of parameters, an improvement in the detection speed, and a simultaneous increase in the detection accuracy of 4.4%.

Method3. adds the D-SimSPPF module as a replacement for the SPPCSPC module seen in the YOLOv7, which was built upon Method2. Due to the implementation of depth-wise separable convolution, the model parameters are decreased to 20.7% of those of YOLOv7, while the number of FLOPs (G) is only 79.2% of that of YOLOv7, effectively achieving lightweighting. Although the accuracy decreased slightly compared to that of Method 2, the mAP of Method 3 was 4.2% higher than that of Method 1. Hence, the efficacy of enhancing the D-SimSPPF module is shown.

Method 4 introduces the SimAM to both the feature fusion network and the head layer. The model's mAP increased to 97% without any increase in its parameter count or computational complexity. This demonstrates that integrating the of SimAM attention mechanism into the framework fulfils the demand for model lightweighting, while simultaneously enabling the merging of feature information at many scales to accomplish the precise identification of defective targets. Compared with Method 1, Method 4 (SS-YOLO) exhibited a 4.5% increase in the mAP. Additionally, the number of FLOPs (G) increased to 21.8, while the number of parameters decreased to 29,501,282.

To summarize, the proposed SS-YOLO network in this paper enhances the mAP while simultaneously minimizing the model parameters and increasing the computational speed. This approach achieves a desirable balance between model accuracy and lightweighting, making it suitable for real-time surface defect detection on strip steel in industrial scenarios.

### Performance comparison of different detection algorithms

To validate the effectiveness of the SS-YOLO algorithm proposed in this paper for detecting surface defects in complex strip steel industrial production scenarios, we conducted comparative experiments between SS-YOLO and several mainstream defect detection algorithms such as Faster R-CNN^11^, SSD^3^, YOLOv3^5^, YOLOv4^6^, YOLOv5^7^ and YOLOv7^9^ on the NEU-DET dataset. The results of the comparison are shown in Table 6.

**Table 6:** Performance comparison of different detection algorithms.

Model	Params (M)	FLOPs (G)	mAP (%)	AP%					
				Cr	In	Pa	Ps	Rs	Sc
Faster R-CNN^11^	56.8	25.3	77.5	33.5	86.3	93.9	90.0	65.4	96.1
SSD^3^	48.8	97.6	62.3	32.7	82.4	71.9	69.6	58.4	78.5
YOLOv3^5^	61.6	193.8	73.4	37.5	74.3	91.3	84.8	70.2	82
YOLOv4^6^	24.4	53.4	80.4	31.7	75.3	81.8	72.4	45.6	78.2
YOLOv5^7^	20.9	45.5	74.6	43.5	69.6	91.5	88.3	66.4	94.4
YOLOv7^9^	37.2	105.2	92.5	90.9	86.9	99.5	94	88.8	95.1
SS-YOLO	29.5	21.8	97	98	93.7	99.7	97.7	96	96.9

Table 6 demonstrates that the SS-YOLO model described in this paper attains the greatest precision in detecting all six defects: CR, In, Pa, Ps, Rs, and Sc.

Despite having a slightly higher parameter count than YOLOv4 and YOLOv5, SS-YOLO still has a competitive advantage. With an enhanced detection speed of 21.8%, surpassing the YOLOv4 and YOLOv5 by 31.6% and 23.7%, respectively. Furthermore, SS-YOLO achieves an impressive 97% detection accuracy, outperforming YOLOv4 and YOLOv5 by 16.6% and 22.4%, respectively. This demonstrates that SS-YOLO exhibits superior detection speed and accuracy, rendering it very compatible for implementation on hardware devices designed for strip surface defect detection.

In terms of detection accuracy, the SS-YOLO model achieves the highest mAP surpassing those of the widely used YOLOv5 algorithm and the high-precision YOLOv7 algorithm by 22.4% and 4.5%, respectively. This finding suggested that the SS-YOLO algorithm has a certain advantage in terms of detection accuracy among target detection algorithms. Through comparative experiments, it is demonstrated that this model has superior detection speed and accuracy, achieving effective lightweighting. This method meets the real-time job requirements for identifying surface defects in steel strips and outperforms other mainstream target detection methods.

**Figure 9:**
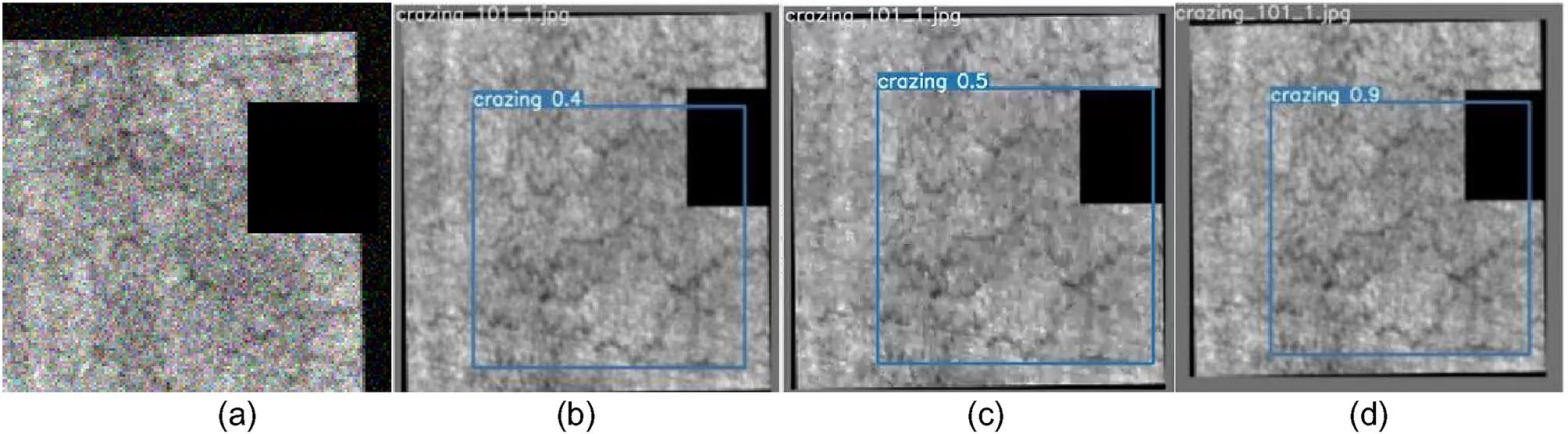
Comparison of crazing defect detection. **(a)** Original images; **(b)** YOLOv5; **(c)** YOLOv7; **(d)** SS-YOLO.

**Figure 10:**
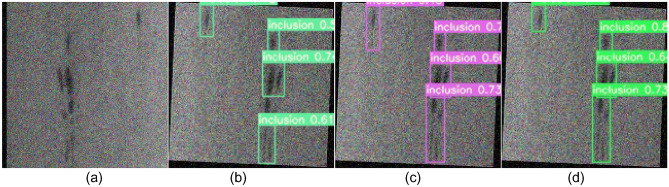
Comparison of inclusion defect detection. **(a)** Original images; **(b)** YOLOv5; **(c)** YOLOv7; **(d)** SS-YOLO.

**Figure 11:**
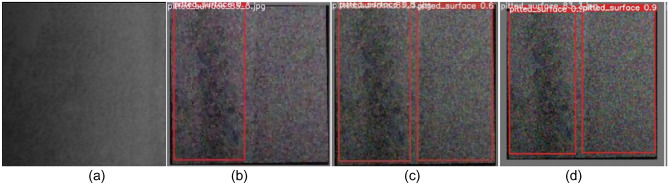
Comparison of pitted_surface defect detection. **(a)** Original images; **(b)** YOLOv5; **(c)** YOLOv7; **(d)** SS-YOLO.

**Figure 12:**
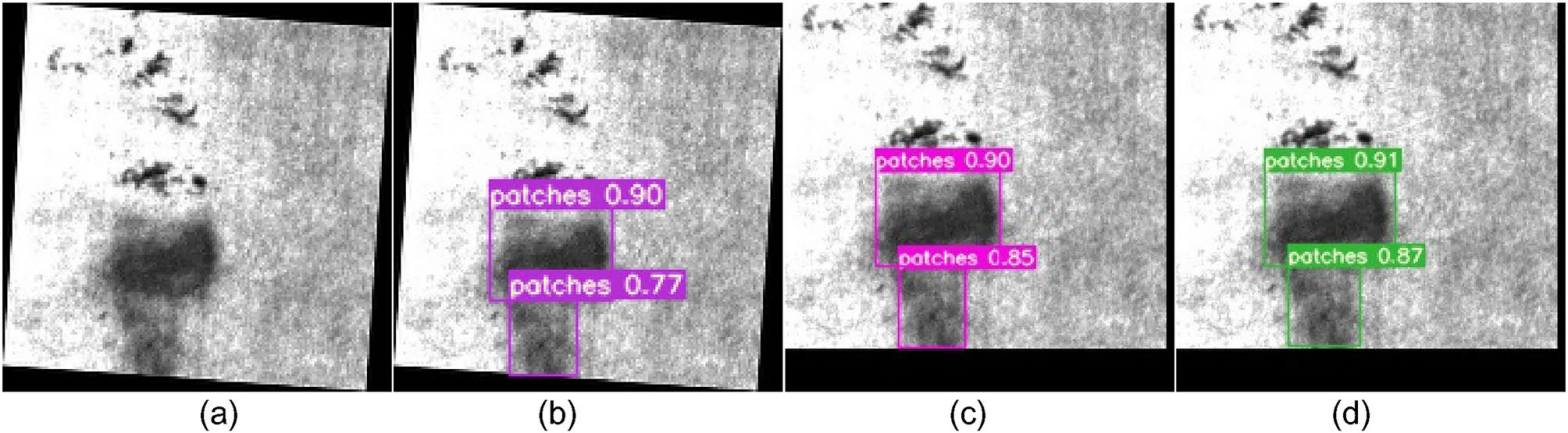
Comparison of patches defect detection. **(a)** Original images; **(b)**YOLOv5; **(c)** YOLOv7; **(d)** SS-YOLO.

**Figure 13:**
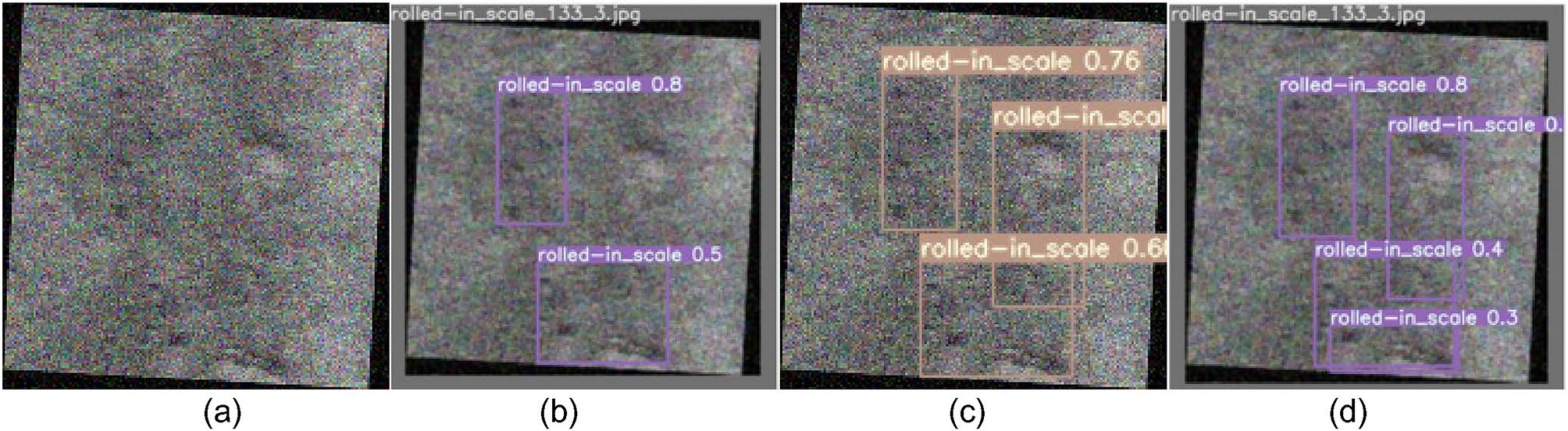
Comparison of rolled-in scale defect detection. **(a)** Original images; **(b)**YOLOv5; **(c)** YOLOv7; **(d)** SS-YOLO.

**Figure 14:**
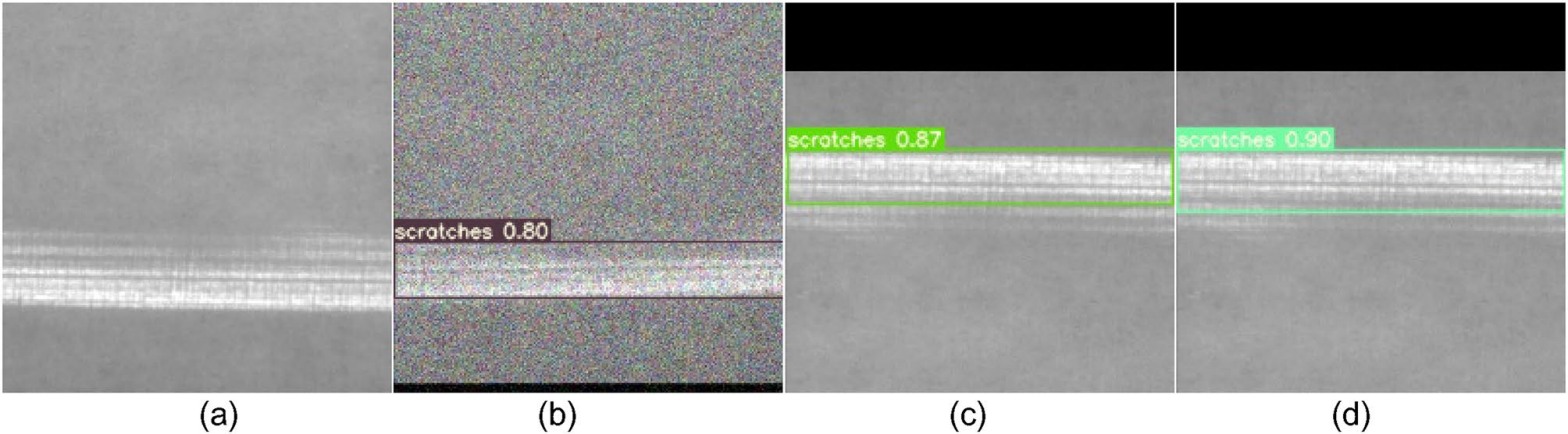
Comparison of scratches defect detection. **(a)** Original images; **(b)** YOLOv5; **(c)** YOLOv7; **(d)** SS-YOLO.

To more clearly demonstrate the improvement in detection effectiveness achieved by the improved SS-YOLO model, we conducted tests using the YOLOv5, YOLOv7, and improved SS-YOLO models on the NEU-DET test dataset. The images display the predicted bounding boxes, defect categories, and confidence scores, with some sample detection results shown in Figs. 9, 10, 11, 12, 13, 14.

Figures 9b, 10, 11, 12, 13, 14b represent the detection results of the YOLOv5 model, showing a total of 3 instances of missed detections (pitted_surface defect detection and rolled-in scale defect detection). Figures 9c, 10, 11, 12, 13, 14c depict the detection results of the YOLOv7 model, with 1 instance of missed detection (rolled-in scale defect detection). Figures 9d, 10, 11, 12, 13, 14d show the detection results of the SS-YOLO model, which did not exhibit any missed detections and displayed higher confidence scores than the YOLOv5 and YOLOv7 models.

By comparing the images, it is evident that the improved SS-YOLO model, compared to the widely used YOLOv5 model and the high-precision YOLOv7 model, achieves higher confidence scores and can more accurately identify defects while also addressing instances of missed defect detection on the surface of steel strips to some extent. Overall, the SS-YOLO model demonstrates outstanding detection performance in real-time defect detection tasks on the surface of steel strips.

## Conclusion

To address the problems of high computational complexity and slow detection speed of the current strip steel surface defect detection model, a lightweight and improved YOLOv7 strip steel surface defect detection method, SS-YOLO, is proposed. The MobileNetv3 network, which is lightweight, serves as the backbone network to decrease the number of model parameters and computational complexity. Additionally, the detection accuracy of the model greatly increases. The purpose of the D-SimSPPF module is to replace the SPPCSPC model, compress network parameters and integrate multiscale feature information. The parameter-free SimAM attention mechanism is presented to improve the model recognition accuracy without adding to the total number of model parameters. Compared to the baseline YOLOv7 model, the lightweight improved SS-YOLO model achieves a detection accuracy of 97% on the NEU-DET dataset, showing an improvement of 4.5%, with 21.8 FLOPs(G), which is only 79.3% that of the basic model. Several comparative experiments show that the proposed SS-YOLO model demands fewer computational resources while preserving high detection accuracy. This approach effectively resolves the trade-off between detection accuracy and speed, making it a competitive solution in strip steel surface defect detection. Subsequent investigations will prioritize reducing of the number of parameters in the model while maintaining high accuracy in detecting surface defects on steel strips. We will continue to optimize improvement tactics to obtain higher detection accuracy and better fulfil the real-time requirements of surface flaw detection on steel strips.

## Data Availability

The data that support the findings of this study are openly available in [NEU-DET] at http://faculty.neu.edu.cn/songkc/en/zhym/263264/list/index.htm.
